# Weight Change and Predictors of Weight Change Among Patients Initiated on Darunavir/Cobicistat/Emtricitabine/Tenofovir Alafenamide or Bictegravir/Emtricitabine/Tenofovir Alafenamide: A Real-World Retrospective Study

**DOI:** 10.36469/001c.24535

**Published:** 2021-06-14

**Authors:** Bruno Emond, Carmine Rossi, Aurélie Côté-Sergent, Keith Dunn, Patrick Lefebvre, Marie-Hélène Lafeuille, Prina Donga

**Affiliations:** 1 Analysis Group, Inc.; 2 Janssen Scientific Affairs, LLC

**Keywords:** observational study, electronic health records, body mass index, weight gain, integrase inhibitors, protease inhibitors, hiv

## Abstract

**Background:** Recent evidence suggests that integrase strand transfer inhibitors are associated with greater weight gain than protease inhibitors in patients with human immunodeficiency virus (HIV-1).

**Objectives:** To describe demographic and clinical characteristics of insured patients with HIV-1 in the United States initiating darunavir/​cobicistat/​emtricitabine/​tenofovir alafenamide (DRV/c/FTC/TAF) or bictegravir/FTC/TAF (BIC/FTC/TAF), assess the differences in weight and body mass index (BMI) change between cohorts up to one year after treatment initiation, and identify the predictors of weight gain associated with each treatment.

**Methods:** The Symphony Health, IDV® database (July 17, 2017 – September 30, 2019) was used to identify treatment naïve or virologically suppressed stable switchers who initiated DRV/c/FTC/TAF or BIC/FTC/TAF (index date) on or after July 17, 2018, were ≥18 years of age on the index date, and had ≥12 months of continuous clinical activity pre-index (baseline period). To account for differences in baseline characteristics, inverse-probability of treatment weighting (IPTW) was used. Mean weight and BMI change from pre- to post-index measurements were compared between weighted cohorts at 3, 6, 9, and 12 months post-index using mean differences. Predictors of weight or BMI gain ≥5% were evaluated at last measurement, for each treatment cohort separately.

**Results:** After IPTW, 452 and 497 patients were included in the DRV/c/FTC/TAF and BIC/FTC/TAF cohorts, respectively. Baseline characteristics were generally well-balanced (mean age=~50 years, female: ~30%), except for the type of antiretroviral therapy from which patients switched. Patients initiated on BIC/FTC/TAF experienced greater weight and BMI increases between the pre-index period and each measurement of the post-index period than patients initiated on DRV/c/FTC/TAF, although results were only statistically significant at 9 months post-index (weight: mean difference=2.50 kg, *P*=0.005; BMI: mean difference=0.66 kg/m^2^, *P*=0.027). A common predictor of weight or BMI gain ≥5% among patients in both cohorts was female gender (DRV/c/FTC/TAF: odds ratio [OR]=5.92, *P*=0.014; BIC/FTC/TAF: OR=2.00, *P*<0.001).

**Conclusion:** Patients in the BIC/FTC/TAF cohort experienced greater weight and BMI increases than patients in the DRV/c/FTC/TAF cohort, with differences reaching statistical significance at 9 months post-index. Weight gain is an important factor to consider when selecting antiretroviral regimens, since it is associated with long-term health consequences. Future studies with larger sample size and longer follow-up time are warranted.

## BACKGROUND

Human immunodeficiency virus (HIV-1) is a chronic infectious disease characterized by a decline in the number of CD4+ T cells, which underlies the immunosuppression observed in affected individuals.^1^ Although HIV-1 cannot be cured, the use of antiretroviral therapy (ART) can effectively reduce the risk of transmission,^2–6^ and improve clinical outcomes^7^ and quality of life.^4^

The United States (US) Department of Health and Human Services (DHHS) guidelines recommend that an integrase strand transfer inhibitor (INSTI)-based ART regimen be used in most clinical situations.^8^ Due to their high genetic barrier to resistance, protease inhibitor (PI)-based regimens are also recommended in patients at risk of non-adherence or among patients who rapidly initiate treatment.^8^ Additionally for those rapidly initiating treatment, a three drug regimen with dolutegravir, bictegravir (BIC), or darunavir (DRV), that does not include abacavir, is recommended.^8^ To facilitate improved adherence, clinicians should also consider single-tablet regimens (STRs), either as initial treatment or to simplify existing treatment regimens. Only two STRs meet these criteria, particularly for rapid initiation. BIC/emtricitabine (FTC)/tenofovir alafenamide (TAF), an INSTI-based STR, was approved on February 7, 2018, while DRV/cobicistat (c)/FTC/TAF, a PI-based STR, was approved on July 17, 2018 in the United States.^9,10^

While the INSTI class had demonstrated a proven tolerability profile, recent evidence suggests that among treatment-naïve patients or stable patients switching from previous ART, those initiated on an INSTI-based regimen were more likely to experience weight gain than those initiated on other types of ART regimens.^11–14^ Recent DHHS guidelines also highlight that greater weight gain is observed among patients treated with INSTI-based regimens relative to PI-based regimens.^8^ Furthermore, INSTI use has been associated with greater risk of metabolic outcomes, including diabetes mellitus.^15–17^ However, no studies to date have assessed weight-related outcomes among patients initiated on DRV/c/FTC/TAF or BIC/FTC/TAF, specifically.

## OBJECTIVES

This real-world study aimed to describe the characteristics of patients initiated on DRV/c/FTC/TAF or BIC/FTC/ TAF in the United States, assess the differences in weight and body mass index (BMI) change at various time points (up to one year after treatment initiation) between patients initiated on those regimens, and identify the predictors of weight gain among patients who initiated each treatment in routine clinical practice.

## METHODS

### Data Source

The Symphony Health, IDV® database with claims and linked electronic medical records (EMR) from July 17, 2017 to September 30, 2019 were used in the current study. The IDV® database links health-care data for the US population from three basic sources: pharmacy point-of-service, switch/network transactions, and additional direct prescription, medical, and hospital claims data. The claims data include pharmacy claims in final form and submitted medical claims, but do not include eligibility records. Claims data capture prescription claims and medical utilization and costs across the United States and covers all payment types, including commercial plans, Medicare Part D, cash, assistance programs, and Medicaid. EMR data includes historical clinical information, such as lab results (CD4/CD8 cell counts and HIV-1 viral loads), and weight and BMI measurements. Data are de-identified and comply with the patient requirements of the Health Insurance Portability and Accountability Act.

### Study Design

A retrospective longitudinal study design was used. The index date was defined as the date of initiation of DRV/c/ FTC/TAF (DRV/c/FTC/TAF cohort) or BIC/FTC/TAF (BIC/FTC/TAF cohort) on or after July 17, 2018 (date of approval of DRV/c/FTC/TAF in the United States). The baseline period was defined as the 12-month period of continuous clinical activity (i.e., the sum of all consecutive quarters during which patients had ≥1 claim in the data) before the index date. The follow-up period spanned from the index date until the earliest of end of continuous clinical activity or end of data availability.

### Study Population

**Figure 1.**
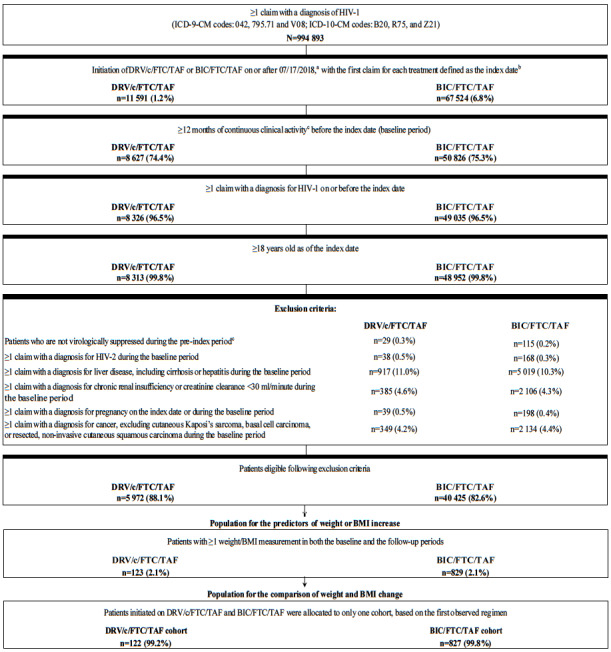
Identification of the Study Population Abbreviations: BIC, bictegravir; BMI, body mass index; c, cobicistat; DRV, darunavir; FTC, emtricitabine; HIV-1, human immunodeficiency virus; ICD-9/10-CM, International Classification of Disease, Ninth/Tenth Revision, Clinical Modification; TAF, tenofovir alafenamide. ^a^Date of approval for DRV/c/FTC/TAF as a single-tablet regimen. ^b^Patients who initiated DRV/c/FTC/TAF and BIC/FTC/TAF were included in both cohorts. ^c^Patients who initiate DRV/c/FTC/TAF and have ≥1 viral load test results ≥50 copies/mL during the 6-month period prior to index date. Patients who initiate BIC/ FTC/TAF and have ≥1 viral load test results ≥50 copies/mL during the 3-month period prior to index date.

Treatment-naïve and previously ART-treated stable (i.e., virologically suppressed) adult patients initiating DRV/c/ FTC/TAF or BIC/FTC/TAF (i.e., first claim defined as the index date) were included in the study population if they additionally had ≥1 claim for an HIV-1 diagnosis on or before the index date, ≥12 months of continuous clinical activity before the index date, and ≥1 weight or BMI measurement in both the baseline and the follow-up periods (Figure 1). Patients were assumed to be treatment-naïve if they had no claim for an ART in the pre-index period, and stable (i.e., virologically suppressed) on a previous ART otherwise, assuming they did not have a viral load test result ≥50 copies/mL. Patients previously treated with an ART who were not stable (i.e., non-virologically suppressed) were excluded from the study. Patients in the DRV/c/FTC/TAF cohort were considered non-virologically supressed if they had ≥1 viral load test result ≥50 copies/mL in the 6-month pre-index period, and those in the BIC/FTC/TAF cohort were considered non-virologically supressed if they had ≥1 viral load test result ≥50 copies/mL in the 3-month pre-index period. The exclusion criterion based on viral load test results was different for each treatment, since it was included to comply with the prescribing guidelines for DRV/c/FTC/TAF and BIC/FTC/TAF, which have different requirements for stable suppressed patients.^9,10^ Additionally, patients were excluded if they had ≥1 claim with a diagnosis for HIV-2, liver disease (including cirrhosis), hepatitis, chronic renal insufficiency (or creatinine clearance <30 mL/minute), or cancer (excluding cutaneous Kaposi’s sarcoma, basal cell carcinoma, or resected, non-invasive cutaneous squamous carcinoma) during the baseline period, or if they had ≥1 claim with a diagnosis for pregnancy on or before the index date.

Of note, the sample selection of patients in the DRV/c/FTC/TAF and BIC/FTC/TAF cohorts was done separately and it was possible for patients to meet the selection criteria for both cohorts. For the comparison of weight-related outcomes between the two cohorts, patients belonging to both cohorts were assigned to only one cohort based on the first of either DRV/c/FTC/TAF or BIC/FTC/TAF that was initiated. To assess predictors of weight gain in each cohort, the independently-derived samples were used.

### Study Measures

Demographic and clinical characteristics were described during the 12-month baseline period. A change in weight or BMI was defined as the difference between the latest pre-index weight or BMI measurement and each available post-index weight or BMI measurements at various time points. The weight or BMI measurement closest to the index date in the baseline period (or within 45 days post-index if no pre-index measurements were available) was defined as the pre-index weight or BMI measurement. The post-index weight or BMI measurements were evaluated at 5 different time points: 3, 6, 9, and 12 months following the index date, as well as at the last available post-index measurement (for the identification of the predictors of weight or BMI increase only). These time points were selected to allow assessment of both shorter and longer term weight and BMI changes.

The weight or BMI measurement closest to the 3-, 6-, 9-, or 12-month mark (and within 45 days before or after the mark) was defined as the 3-, 6-, 9-, or 12-month post-index measurement, respectively. Mean weight and BMI change was evaluated at 3, 6, 9, and 12 months post-index, and defined as the mean difference between the post- and the pre-index measurement. Similarly, the proportion of patients having any, ≥5%, and ≥10% weight and BMI increase was evaluated at 3, 6, 9, and 12 months post-index, and was defined as having an increase between the post- and the pre-index measurement >0%, ≥5%, and ≥10%, respectively. For the identification of the predictors of weight or BMI increase, ≥5% weight or BMI increase was evaluated at the last available post-index measurement and defined as having an increase between the post- and the pre-index measurement ≥5%.

### Statistical Analysis

Baseline characteristics were reported using means, standard deviations (SDs), and medians for continuous variables, and counts and proportions for categorical variables. Inverse probability of treatment weighting (IPTW) based on propensity scores (PSs) was used to account for differences in baseline characteristics between treatment cohorts. The PS for each patient was estimated using a multivariable logistic regression model adjusting for the following baseline covariates: age, gender, race, region, insurance plan/payer type, year of the index date, presence of substance-related and addictive disorders, hypertension, type 2 diabetes, obesity, Quan-Charlson Comorbidity index (Quan-CCI), and BMI. Each patient was assigned a weight of 1/PS for those in the DRV/c/FTC/TAF cohort and 1/(1-PS) for those in the BIC/FTC/TAF cohort; weights were then truncated at the 95^th^ percentile (to avoid extreme weights) and normalized by the mean weight. Consequently, the weighted sample sizes (i.e., post-IPTW) were different from the original sample sizes although the same patients contributed to the analysis. In other words, before IPTW, each patient had a weight of one and after IPTW, each patient had a different weight. When adding the weight for each patient after IPTW in a given cohort, the sum of weights (i.e., weighted sample size) was different than the original sample size for a given cohort. The resulting differences between the weighted cohorts of interest reflected the average treatment effect.

The balance of characteristics between the cohorts was assessed using standardized differences (S_diff;_ <10% was considered well-balanced).^18^ The mean change in weight and BMI between the pre- and post-index periods was compared between cohorts at 3, 6, 9, and 12 months post-index using mean differences obtained from ordinary least squares (OLS) regression models. Similarly, the proportion of patients having any, ≥5%, and ≥10% weight and BMI increase between the pre- and post-index periods was compared between cohorts at 3, 6, 9, and 12 months post-index using odds ratios (ORs) obtained from logistic regression models. The OLS and logistic regression models were estimated using a doubly-robust approach, by including baseline covariates in the outcome regression model identical to those included in the PS model. *P*﻿-﻿values and 95% confidence intervals (CIs) were obtained from doubly-robust models. Of note, not all patients had a weight or BMI measurement at all time points; therefore, the number of patients available for comparisons at each time point varied depending on the time point considered.

Additionally, to better understand the factors associated with weight or BMI change in each cohort separately, multivariable logistic regression models were used to identify baseline predictors of having a weight or BMI increase ≥5% at last available measurement. Unweighted results were reported using ORs, 95% CIs, and *P*-values, and a single regression model was evaluated for each cohort (the dependent variable was equal to 1 if patients had either a ≥5% weight increase or a ≥5% BMI increase).

## RESULTS

### Patient Characteristics

A total of 123 and 829 patients were initiated on DRV/c/FTC/TAF and BIC/FTC/TAF, respectively (sample size for predictive analyses), with 122 and 827 patients initiated on DRV/c/FTC/TAF and BIC/FTC/TAF on the index date for the comparative analyses. After weighting the cohorts for the comparative analysis using IPTW, the sum of weights for patients included in each cohort corresponded to weighted sample sizes of 452 patients in the DRV/c/FTC/TAF cohort and 497 patients in the BIC/FTC/TAF cohort. Baseline characteristics included in the PS model were generally well-balanced, with the exception of one US geographic region (i.e., Midwest region) and two insurance plan/payer categories (i.e., commercial plans and assistance programs; Table 1). Mean age was 53.1 years (SD=11.2) in the DRV/c/FTC/TAF cohort and 52.2 years (SD=12.0) in the BIC/FTC/TAF cohort; 30.0% and 28.2% of patients in the DRV/c/FTC/TAF and BIC/FTC/TAF cohort were female, respectively. Most patients in both cohorts were white (DRV/c/FTC/TAF: 31.9%; BIC/FTC/TAF: 35.8%) or Black/African American (DRV/c/FTC/TAF: 30.6%; BIC/FTC/TAF: 27.2%), and were covered by commercial plans (DRV/c/FTC/TAF: 37.4%; BIC/FTC/TAF: 44.4%) or Medicare (DRV/c/FTC/TAF: 28.3%; BIC/FTC/TAF: 26.9%). The mean baseline Quan-CCI score was 5.0 (SD=2.9) in the DRV/c/FTC/TAF cohort and 4.9 (SD=3.0) in the BIC/FTC/TAF cohort; when excluding HIV-1 symptoms, the Quan-CCI score fell to 0.5 (SD=0.9) and 0.6 (SD=0.9) in each treatment cohort, respectively.

**Table 1.** Baseline Characteristics During the 12-month Period Prior to the Index Date

	**Unweighted Population**			Weighted Population*		
	**DRV/c/FTC/TAF Cohort (n=122)**	**BIC/FTC/TAF Cohort (n=827)**	**Standardized Difference**	**DRV/c/FTC/TAF Cohort (Weighted n=452)**	**BIC/FTC/TAF Cohort (Weighted n=497)**	**Standardized Difference**
Variables Included in the PS Model*						
**Age (years), Mean ± SD [median]**	52.3 ± 10.5 [53.5]	52.2 ± 12.1 [54.0]	1.4%	53.1 ± 11.2 [54.0]	52.2 ± 12.0 [54.0]	8.0%
**Female, n (%)**	32 (26.2)	234 (28.3)	4.6%	136 (30.0)	140 (28.2)	4.0%
**Race, n (%)**						
White	41 (33.6)	299 (36.2)	5.3%	144 (31.9)	178 (35.8)	8.2%
Black/African American	36 (29.5)	221 (26.7)	6.2%	138 (30.6)	135 (27.2)	7.7%
Hispanic	13 (10.7)	66 (8.0)	9.2%	40 (9.0)	41 (8.3)	2.5%
Other	1 (0.8)	15 (1.8)	8.7%	7 (1.5)	9 (1.8)	1.9%
Unknown	31 (25.4)	226 (27.3)	4.4%	122 (27.0)	134 (27.0)	0.1%
**US Geographic Region, n (%)**						
South	73 (59.8)	542 (65.5)	11.8%	292 (64.7)	323 (65.1)	0.8%
West	24 (19.7)	81 (9.8)	28.1%	63 (13.9)	54 (10.8)	9.6%
Northeast	18 (14.8)	96 (11.6)	9.3%	60 (13.3)	60 (12.0)	4.0%
Midwest	7 (5.7)	107 (12.9)	24.9%	36 (8.1)	60 (12.1)	13.3%
Unknown	0 (0.0)	1 (0.1)	4.9%	0 (0.0)	1 (0.1)	4.8%
**Insurance Plan/Payer Type, n (%)**						
Medicare	38 (31.1)	218 (26.4)	10.6%	128 (28.3)	134 (26.9)	3.3%
Medicaid	37 (30.3)	143 (17.3)	31.0%	92 (20.4)	93 (18.8)	4.2%
Commercial Plans	35 (28.7)	384 (46.4)	37.3%	169 (37.4)	221 (44.4)	14.3%
Assistance Programs	12 (9.8)	81 (9.8)	0.1%	63 (13.9)	49 (9.9)	12.3%
Cash	0 (0.0)	1 (0.1)	4.9%	0 (0.0)	1 (0.1)	4.8%
**Year of Index Date, n (%)**						
2018	48 (39.3)	406 (49.1)	19.7%	195 (43.2)	238 (48.0)	9.5%
2019	74 (60.7)	421 (50.9)	19.7%	257 (56.8)	259 (52.0)	9.5%
**Comorbidities, n (%)**						
Substance-related and Addictive Disorders	26 (21.3)	106 (12.8)	22.7%	72 (16.0)	68 (13.7)	6.4%
Hypertension	42 (34.4)	293 (35.4)	2.1%	155 (34.3)	176 (35.4)	2.2%
Type 2 Diabetes Mellitus	15 (12.3)	134 (16.2)	11.2%	70 (15.6)	79 (15.9)	0.8%
Obesity	12 (9.8)	142 (17.2)	21.6%	73 (16.0)	81 (16.4)	0.9%
**Quan-CCI, Mean ± SD [median]**	5.3 ± 2.7 [6.0]	4.8 ± 3.0 [6.0]	16.6%	5.0 ± 2.9 [6.0]	4.9 ± 3.0 [6.0]	5.2%
**Patients with a BMI Measurement, n (%)**	122 (100.0)	817 (98.8)	15.6%	452 (100.0)	491 (98.7)	16.0%
BMI (kg/m^2^), Mean ± SD [median]	28.5 ± 7.1 [27.6]	29.5 ± 6.7 [28.4]	13.9%	28.8 ± 7.2 [28.2]	29.4 ± 6.7 [28.3]	9.6%
BMI Categories (kg/m^2^), n (%)						
<25	38 (31.1)	234 (28.6)	5.5%	137 (30.3)	141 (28.7)	3.5%
≥25 to <30	44 (36.1)	247 (30.2)	12.4%	143 (31.5)	152 (30.9)	1.4%
≥30 to <35	17 (13.9)	182 (22.3)	21.8%	86 (19.0)	105 (21.4)	6.0%
≥35	23 (18.9)	154 (18.8)	0.0%	86 (19.1)	93 (19.0)	0.4%
**Variables Not Included in the PS Model**						
**Comorbidities, n (%)**						
Anxiety Disorders	19 (15.6)	114 (13.8)	5.1%	70 (15.5)	70 (14.0)	4.2%
Depression	28 (23.0)	129 (15.6)	18.7%	98 (21.6)	79 (15.9)	14.7%
Chronic Pulmonary Disease	21 (17.2)	131 (15.8)	3.7%	74 (16.4)	82 (16.4)	0.1%
Drug Abuse	12 (9.8)	47 (5.7)	15.6%	31 (6.9)	31 (6.2)	2.7%
Pre-diabetes	5 (4.1)	32 (3.9)	1.2%	22 (5.0)	19 (3.8)	5.6%
Psychoses	8 (6.6)	50 (6.0)	2.1%	23 (5.2)	32 (6.4)	5.4%
**Quan-CCI (excluding HIV-1 symptoms), Mean ± SD [median]**	0.5 ± 0.9 [0.0]	0.6 ± 0.9 [0.0]	10.7%	0.5 ± 0.9 [0.0]	0.6 ± 0.9 [0.0]	13.3%
**Patients with a Weight Measurement, n (%)**	121 (99.2)	822 (99.4)	2.6%	449 (99.3)	494 (99.4)	0.3%
Weight (kg), Mean ± SD [median]	87.2 ± 22.8 [85.3]	88.3 ± 21.5 [85.3]	5.1%	87.1 ± 22.9 [88.0]	88.2 ± 21.5 [85.3]	5.0%
**Patients with an Absolute CD4 Cell Count Measurement, n (%)**	25 (20.5)	241 (29.1)	20.1%	96 (21.2)	145 (29.2)	18.6%
CD4 Cell Count (cells/µL), Mean ± SD [median]	639.3 ± 362.9 [585.0]	745.9 ± 366.4 [684.0]	29.2%	714.5 ± 405.4 [615.0]	747.0 ± 367.1 [684.0]	8.4%
**Patients with an Absolute CD8 Cell Count Measurement, n (%)**	13 (10.7)	127 (15.4)	14.0%	45 (10.1)	76 (15.2)	15.6%
CD8 Cell Count (cells/µL), Mean ± SD [median]	933.7 ± 555.6 [790.0]	901.5 ± 417.7 [816.0]	6.6%	896.0 ± 557.3 [764.0]	904.8 ± 419.1 [818.0]	1.8%
**Patients with a HIV-1 Viral Load Measurement, n (%)**	51 (41.8)	291 (35.2)	13.6%	183 (40.5)	177 (35.6)	10.0%
HIV-1 Viral Load (copies/mL), Mean ± SD [median]	13.4 ± 12.6 [20.0]	4180.7 ± 29 430.1 [20.0]	20.0%	14.5 ± 13.5 [20.0]	4021.7 ± 28 893.7 [20.0]	19.6%
**Patients with HIV- 1 Disease Onset Information in EMR Data, n (%)**	58 (47.5)	334 (40.4)	14.5%	242 (53.6)	200 (40.3)	27.0%
Time (in months) Between HIV-1 Disease Onset and Index Date, Mean ± SD [median]	147.3 ± 131.4 [87.4]	118.3 ± 224.0 [71.7]	15.8%	129.1 ± 123.4 [85.8]	118.8 ± 222.4 [72.1]	5.7%
**Patients who Switched from a Previous ART to the Index Regimen During the Last 45 Days, n (%)**	92 (75.4)	537 (64.9)	23.1%	332 (73.5)	323 (65.0)	18.5%
Patients who Switched from a PI	73 (59.8)	83 (10.0)	122.5%	258 (57.1)	51 (10.2)	114.3%
DRV-based	67 (54.9)	39 (4.7)	131.3%	244 (54.0)	24 (4.9)	128.1%
Other PI-based	6 (4.9)	44 (5.3)	1.8%	14 (3.0)	27 (5.3)	11.5%
Patients who Switched from an INSTI	26 (21.3)	297 (35.9)	32.7%	101 (22.4)	179 (36.1)	30.4%
Dolutegravir-based	16 (13.1)	108 (13.1)	0.2%	68 (15.1)	66 (13.3)	5.3%
Elvitegravir-based	4 (3.3)	161 (19.5)	52.7%	13 (2.8)	96 (19.3)	54.8%
Raltegravir-based	5 (4.1)	28 (3.4)	3.8%	19 (4.1)	17 (3.5)	3.5%
Bictegravir-based	1 (0.8)	0 (0.0)	12.9%	2 (0.4)	0 (0.0)	8.9%
Patients who Switched from an NNRTI	2 (1.6)	165 (20.0)	61.8%	7 (1.5)	99 (19.9)	62.1%
**NRTIs Used +/- 14 days from the Most Recent Switch, Among Switchers n (%)**						
FTC/TAF	65 (70.7)	267 (49.7)	43.8%	216 (65.1)	162 (50.2)	30.5%
FTC/TDF	10 (10.9)	171 (31.8)	52.9%	43 (13.0)	101 (31.3)	45.3%
Abacavir/lamivudine	7 (7.6)	54 (10.1)	8.6%	36 (10.8)	33 (10.2)	2.0%

Abbreviations: ART, antiretroviral therapy; BIC, bictegravir; BMI, body mass index; c, cobicistat; CCI, Charlson Comorbidity Index; DRV, darunavir; EMR, electronic medical records; FTC, emtricitabine; HIV-1, human immunodeficiency virus; INSTI, integrase strand transfer inhibitor; NNRTI, non-nucleoside reverse transcriptase inhibitor; NRTI, nucleoside/nucleotide reverse transcriptase inhibitors; PI, protease inhibitor; PS, propensity score; SD, standard deviation; TAF, tenofovir alafenamide; TDF, tenofovir disoproxil fumarate; US, United States.

*Inverse probability of treatment weighting (IPTW) based on propensity scores (PSs) was used to account for differences in baseline characteristics between treatment cohorts. The PS for each patient was estimated using a multivariable logistic regression model adjusting for the following baseline covariates: age, gender, race, region, insurance plan/payer type, year of the index date, presence of substance-related and addictive disorders, hypertension, type 2 diabetes, and obesity, Quan-CCI, and BMI. Age was modeled using both continuous and categorical variables. With the exception of Quan-CCI, which was modeled as a continuous variable, the other remaining covariates were modeled using categorical variables.

The mean baseline BMI in the DRV/c/FTC/TAF and BIC/FTC/TAF cohorts was 28.8 kg/m^2^ (SD=7.2) and 29.4 kg/m^2^ (SD=6.7), respectively, while the mean baseline weight was 87.1 kg (SD=22.9) and 88.2 kg (SD=21.5), respectively (Table 1). The proportion of patients who switched from a previous ART to the index regimen during the last 45 days prior to the index was higher in the DRV/c/FTC/TAF cohort than in the BIC/FTC/TAF cohort (73.5% vs. 65.0%, S^diff^=18.5%). In the DRV/c/FTC/TAF cohort, 57.1% of patients switched from a PI-based regimen (including 54.0% switching from a DRV-based multiple-tablet regimen and 3.0% switching from another PI-based regimen), 22.4% switched from an INSTI-based regimen, and 1.5% switched from a non-nucleoside reverse transcriptase inhibitors (NNRTI)-based regimen, while in the BIC/FTC/TAF cohort, 36.1%, 19.9%, and 10.2% of patients switched from an INSTI-, NNRTI-, and PI-based regimen, respectively. Only 4.9% of patients in the BIC/ FTC/TAF cohort switched from a DRV-based regimen. Among switchers, in the DRV/c/FTC/TAF cohort, 65.1% of patients had a claim for FTC/TAF within 14 days of the most recent ART switch, which was higher than in the BIC/FTC/TAF (50.2%, S^diff^=30.5%).

### Comparison of Weight and BMI Change

**Figure 2.**
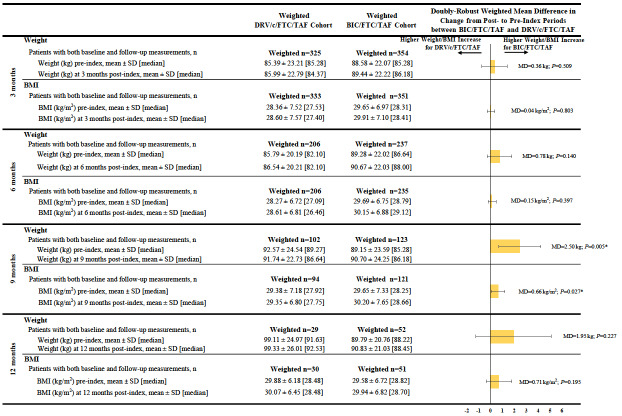
Mean Change in Weight and BMI Between the Pre- and Post-index Periods Abbreviations: BIC, bictegravir; BMI, body-mass index; c, cobicistat; CI, confidence interval; DRV, darunavir; FTC, emtricit- abine; MD, mean difference; SD, standard deviation; TAF, tenofovir alafenamide. *Indicates *P*-value<0.05.

Patients in the BIC/FTC/TAF cohort experienced a greater weight increase between the pre- and post-index periods than patients in the DRV/c/FTC/TAF cohort across all time points, although results were only statistically significant at the 9-month post-index time point (mean difference at 9 months=2.50 kg, *P*=0.005). Similar results were found for BMI increases between the pre- and post-index periods (mean difference at 9 months=0.66 kg/m^2^, *P*=0.027; Figure 2).

**Figure 3.**
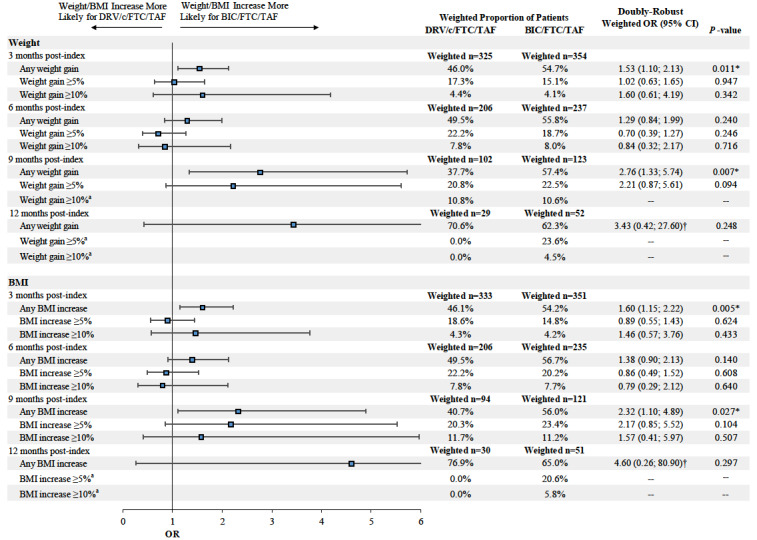
Proportions of Patients Having any, ≥5%, and ≥10% Weight or BMI Increases Between the Pre- and Post-index Periods Abbreviations: BIC, bictegravir; BMI, body mass index; c, cobicistat; CI, confidence interval; DRV, darunavir; FTC, emtricitabine; OR, odds ratio; TAF, tenofovir alafenamide. *Indicates *P*-value<0.05. †Indicates that the right bound of the 95% CI exceeds the range of the horizontal axis. ^a^The OR was not reported due to the small sample size and large number of covariates included in the model, which resulted in lack of model convergence.

Furthermore, at 9 months post-index, the proportion of patients experiencing any weight or BMI increase (i.e., weight or BMI increase >0%) was higher among patients initiated on BIC/FTC/TAF compared to those initiated on DRV/c/FTC/TAF (weight increase: 57.4% vs 37.7%, OR=2.76, *P*=0.007; BMI increase: 56.0% vs 40.7%, OR=2.32, *P*=0.027). Similar findings were found for weight increase ≥5% and BMI increase ≥5% and ≥10% at 9 months post-index, although results did not reach statistical significance (the OR for a weight increase ≥10% was not evaluated due to the small sample size and large number of covariates included in the model, which resulted in lack of model convergence; Figure 3).

At 3, 6, and 12 months post-index, the proportion of patients experiencing any, ≥5%, and ≥10% weight and BMI increase was not statistically different between patients in the DRV/c/FTC/TAF and BIC/FTC/TAF cohorts, with the exception of the proportion of patients experiencing any weight or BMI increase at 3 months post-index, which was higher among patients initiated on BIC/FTC/TAF compared to those initiated on DRV/c/FTC/TAF (Figure 3).

### Predictors of Weight or BMI Increase ≥5%

Among patients initiated on DRV/c/FTC/TAF eligible for the predictive analysis (n=123, Figure 1), female gender was associated with a higher likelihood of weight or BMI increase ≥5% (OR=5.92, *P*=0.014) at the last available measurement post-index, while higher baseline BMI (i.e., ≥25 kg/m^2^) was associated with a lower likelihood of weight or BMI increase ≥5% (OR=0.27, *P*=0.028; Figure 4A). Among patients initiated on BIC/FTC/TAF eligible for the predictive analysis (n=829, Figure 1), female gender was also associated with a higher likelihood of weight or BMI increase ≥5% (OR=2.00, *P*<0.001), while a 5-year increase in age (OR=0.87, *P*=0.001) and the use of an INSTI-based regimen in the pre-index period (OR=0.62, *P*=0.022) were associated with a lower likelihood of weight or BMI increase ≥5% (Figure 4B).

**Figure 4a.**
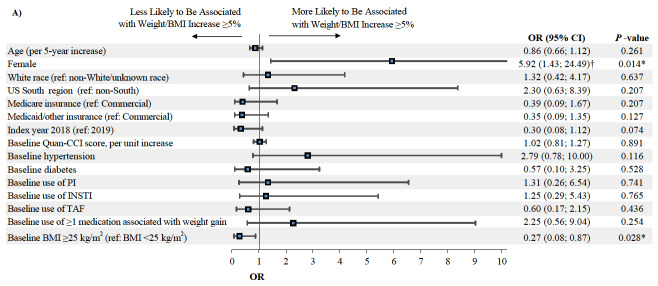
Predictors of Weight or BMI Increase ≥5% Among Patients Initiating DRV/c/FTC/TAF Abbreviations: BIC, bictegravir; BMI, body mass index; c, cobicistat; CCI, Charlson Comorbidity Index; CI, confidence interval; DRV, darunavir; FTC, emtricitabine; INSTI, integrase strand transfer inhibitor; OR, odds ratio; PI, protease inhibitor; TAF, tenofovir alafenamide; US, United States. *Indicates *P*-value<0.05. †Indicates that the right bound of the 95% CI exceeds the range of the horizontal axis.

**Figure 4b.**
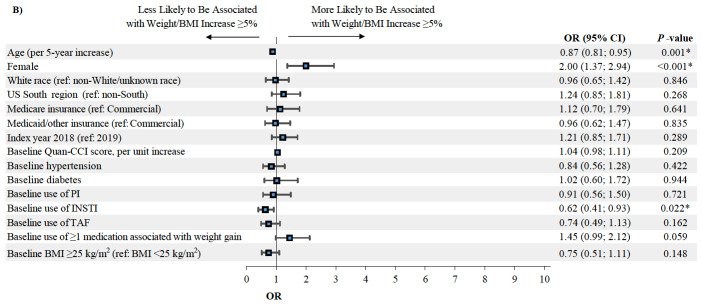
Predictors of Weight or BMI Increase ≥5% Among Patients Initiating BIC/FTC/TAF Abbreviations: BIC, bictegravir; BMI, body mass index; c, cobicistat; CCI, Charlson Comorbidity Index; CI, confidence interval; DRV, darunavir; FTC, emtricitabine; INSTI, integrase strand transfer inhibitor; OR, odds ratio; PI, protease inhibitor; TAF, tenofovir alafenamide; US, United States. *Indicates *P*-value<0.05. †Indicates that the right bound of the 95% CI exceeds the range of the horizontal axis.

## DISCUSSION

This was the first real-world study to use linked claims and EMR data to examine real-world weight and BMI changes in treatment-naïve and stable patients previously treated with an ART who were initiated on DRV/c/FTC/ TAF, a PI-based STR, or BIC/FTC/TAF, an INSTI-based STR. Results showed that among a diverse population of HIV-1 patients, those initiated on BIC/FTC/TAF experienced greater weight and BMI increases between the pre-index period and each measurement of the post-index period than patients initiated on DRV/c/FTC/TAF, although results were only statistically significant at the 9-month post-index time point (mean difference in weight gain=2.50 kg [5.5 lbs]; mean difference in BMI increase=0.66 kg/m^2^). In addition, predictive analyses showed that a common predictor of weight or BMI gain ≥5% among patients in both cohorts was female gender.

The 9-month post-index time point may be an interesting time point to focus on in the current study, since it can be considered as a long-term indicator of weight gain compared to the 3- and 6-month time points (i.e., short-term time points) and since the 12-month post-index time point lacked power due to sample sizes that were too small to adequately evaluate differences between cohorts. Indeed, this study showed that mean differences in weight and BMI between treatment cohorts generally increased over time, which is consistent with a prior study in which patients experienced weight gain for up to 96 weeks following ART initiation, with the most important weight gain occurring in the first 48 weeks.^19^ Since patients may continue to experience weight gain over time following treatment initiation, it is likely that at the 3 and 6 months post-index time points, they were experiencing smaller increases in their weight and BMI, which may explain why results were not yet statistically significant.

Although a moderate weight increase among patients with advanced HIV-1 may be considered favorable and expected (i.e., a manifestation of the return to health phenomenon),^19^ prior research has demonstrated an increasing prevalence of obesity and weight gain among people living with HIV-1 (PLWH) who do not have advanced disease and are simply initiating ART.^20–22^ Notably, PLWH have been shown to carry a higher risk of comorbid conditions, such as cardiovascular disease,^23,24^ which is magnified by weight gain following ART initiation, increasing the risk of diabetes and cardiovascular diseases.^25–28^ For example, it has previously been reported that among patients initiating ARTs, every incremental weight gain of 5 lbs was associated with 14% increased risk of incident diabetes and a unit increase of BMI was associated with 20% increased risk of cardiovascular diseases.^27,29^ Additionally, almost half of PLWH in the United States are 50 years or older,^29^ and as the treatment landscape for HIV-1 continues to evolve, the number of PLWH over 50 years will continue to increase, placing older PLWH at an increased risk for comorbid age-associated health conditions, such as diabetes mellitus, hypertension, and cardiovascular disease.^30,31^ Although the weight and BMI increases observed in this study were small, the potential for cumulative gains could be considerable over time. Therefore, it is vital to address issues of treatment-related weight and BMI gain among PLWH initiating ARTs to mitigate the long-term health consequences, while balancing other considerations that may guide the choice of ART, such as adherence to treatment.

This study reflects real-world prescribing practices for DRV/c/FTC/TAF and BIC/FTC/TAF regimens and as a result, differences in baseline demographic and clinical characteristics between treatment cohorts were observed. Some baseline characteristics between treatment cohorts remained imbalanced despite the use of IPTW, particularly in terms of the type of ART patients switched from during the last 45 days prior to the index date. Given that DRV/c/FTC/TAF and BIC/FTC/TAF regimens were approved in 2018, this real-world study identified a large proportion of previously ART-treated patients during the 2017 to 2019 period. Nearly two-thirds of patients in the DRV/c/FTC/TAF cohort switched to the index regimen from a PI-based regimen (nearly all were DRV-based), which is in line with DHHS guidelines that recommend within-class switches when simplifying treatment regimens.^8^ A different pattern was observed in the BIC/FTC/TAF cohort, where one-third of patients switched to the index regimen from an INSTI-based regimen (most commonly elvitegravir-based) and only 4.9% switched to the index regimen from a DRV-based regimen. Importantly, a larger proportion of patients in the DRV/c/FTC/TAF cohort than in the BIC/FTC/TAF cohort switched from a regimen containing TAF, which has been associated with weight increase in other studies.^19,32–35^ Furthermore, a smaller proportion of patients in the DRV/c/FTC/TAF cohort switched from a regimen containing tenofovir disoproxil fumarate (TDF), which has been associated with weight suppression.^36^ The inability to adjust for these prior ART-related imbalances (e.g., inability to adjust for the previous switch from a PI or INSTI and for the previous use of TAF or TDF) was the result of a small sample size stemming from the necessity to link claims to EMR data to obtain weight and BMI measurements, thus limiting the number of variables that could be included in the propensity score model for the IPTW. Consequently, important ART-related confounding factors may have had an impact on the results observed in the current analysis. The use of IPTW possibly mitigated this limitation as the resulting pre-index weight and BMI were relatively similar between cohorts. In addition, a descriptive analysis of weight and BMI measurements between patients in the DRV/c/FTC/TAF cohort who switched from a DRV-based regimen and those who switched from another type of ART regimen showed that no clear trends in weight or BMI increase could be identified between these two groups of patients (results not shown). However, future studies with larger sample sizes and longer follow-up times that allow for the adjustment of additional confounders such as the type of ART patients switch from are warranted to better assess the differences in weight and BMI change between patients initiating DRV/c/FTC/TAF or BIC/FTC/TAF.

In addition to differences in previous treatments used, INSTI-based regimens (including BIC/FTC/TAF) are recommended by the DHHS guidelines as initial treatments for most patients with HIV-1,^8^ while PI-based regimens (including DRV/c/FTC/TAF) are recommended only in certain clinical situations (such as when rapid initiation is needed in the absence of available resistance testing results). Therefore, channeling bias may have been present in the current study. Indeed, otherwise healthier patients are likely channeled to being prescribed INSTI-based regimens instead of PI-based regimens. Although not completely eliminated, this type of bias was mitigated by adjusting for observable differences in patient characteristics (including comorbidities) between patients initiating DRV/c/FTC/TAF and BIC/FTC/TAF.

Although predictors of weight or BMI increase ≥5% varied for each treatment cohort, female gender was a common factor identified, in line with previous findings and DHHS guidelines.^8,19,37^ Despite these differences in weight gain between genders, a prior study of virologically-suppressed patients previously treated with a boosted PI and switching to DRV/c/FTC/TAF found that other outcomes (such as the proportion of patients experiencing virologic rebound or patient adherence to medication) were similar between males and females.^38^ Similar to the assessment of weight and BMI change, the identification of predictors of weight or BMI increase ≥5% in both cohorts was also limited by a small sample size, resulting in the inclusion of a limited number of predicting factors in the models.

This study is subject to certain limitations beyond those associated with prior ART-related imbalances between treatment cohorts (including the inability to adjust for previous use of PI, INSTI, or TAF) and small sample sizes, which were discussed above. First, ART claims are assumed to indicate their use; however, patients may not adhere to the treatment regimen as prescribed. Additionally, as with all claims and EMR data sources, the Symphony Health, IDV^®^ claims and EMR data may contain inaccuracies or omissions in diagnoses, billing, and other variables such as date of service, days of supply, and recorded weight or BMI, CD4^+^ cell counts, and HIV-1 viral load measurements.

## CONCLUSIONS

Greater weight gain and BMI increases were observed among patients initiated on BIC/FTC/TAF relative to those initiated on DRV/c/FTC/TAF, with results reaching statistical significance at 9 months post-index and with mean differences between cohorts generally increasing over time. Female gender was associated with a higher likelihood of weight or BMI increase ≥5% among both the DRV/c/FTC/TAF and BIC/FTC/TAF cohorts. Future studies with larger sample sizes and longer follow-up times that allow for the adjustment of additional confounders (such as the type of ART patients switch from) or among treatment-naïve patients are warranted to better assess the differences in weight and BMI change between PLWH initiating DRV/c/FTC/TAF or BIC/FTC/TAF.
